# Some Historic Points and Practical Hints on Crowning

**Published:** 1890-10

**Authors:** Thos. G. Read

**Affiliations:** HARVARD.


ARTICLE V.

### SOME HISTORIC POINTS AND PRACTICAL HINTS ON CROWNING.

BY THOS. G. READ, L. D. S., ENG., D.M.D., HARVARD.

A paper read before the Students Society, National Dental Hospital, and published in the *British Journal of Dental Science*.

The clinical aspect of this subject as far as possible, I shall avoid, therefore the making of any special crown will not be described.

Crowning was probably attempted at a very early date.

In former days teeth required to be very loose to be removed, as the ancients had no suitable instruments with which to extract, and there was a strong feeling against mutilating the body. The hard work that the anterior teeth had to perform in tearing flesh from bones and other uses, to which they were put, moreover their exposed position renders them very liable to be broken.

No doubt some effort was made to correct this disfigurement, and it may be readily conceived that when an opening was noticed in the broken off part to correspond to one in the remains of the tooth left in the jaw, the broken or the corresponding part of another tooth was connected to the stump by means of a dowel or pin, often miscalled a pivot.

The earliest crowns were portions of natural teeth both of human beings and of the lower animals and artificial ones carved from various tooth-like substances.

I shall endeavor to conclusively prove to you that all crowns now used are modifications of methods known and practiced forty years ago.

In the year 1774, M. Duchateau, an apothecary of St. Germaine, and M. de Chemart, a dentist of paris, commenced experiments with the object of making teeth of porcelain, and after several years of perseverance they produced a material, the merits of which were fully recognized by the profession for dental uses.

Crowns were soon made in this material, they roughly resembled teeth and had a hold for a dowel, they were rather weak, being made of a poor kind of porcelain resembling wedgewood were glazed over with a semi-transparant glass of a greenish or blueish tint, the dowel used to set them with was usually of wood alone, or wood and wire.

But little improvement was made in their appearance until the year 1825, when Samuel W. Stockton, of Philadelphia, and a few others made a better composition and produced teeth much more life-like in shape and shade; in 1830 they made some crowns which were in many respects better adapted for use than any of a later date; the dowel hole was deeper, of better form and more conveniently placed.

In 1831, Dr. Lee of Alabama, used instruments very much like those described and used later by Dr. Brutter, these are of very little use as they are only suitable for circular roots.

In 1841, tube teeth with a metal dowel were used.

In 1849, a hollow dowel was patented for permitting gases and secretions to escape from the apex of the root.

In the same year, Dr. Lawrence, of Philadelphia, patented a crown similar to the Bonwill, it consisted of porcelain with a counter-sunk aperture for a screw.

Dr. Morrison, about this time, used a crown very like a Richmond. In describing this he mentioned a band or collar fitting around the root supporting a crown with a dowel. The logan is a modification of the old porcelain wood dowel crown, platinum being used instead of wood.

An ideal crown should reduce the chance of destruction of tooth substance to the minimum, be non-irritant to adjacent tissues, natural in appearance, strong enough to resist the strain of mastication and capable of being quickly constructed.

The only apparent manner in which a stump that is properly crowned can be lost is by disease in connection with the periosteum.

Crowning should not be attempted when the tooth is very loose, necrosed, exostosed, or surrounded with unhealthy gum.

It will be found impossible to adapt a collar or cap a crown in cases where the natural crown has been broken away for some time and the adjacent teeth have moved so as to form a triangular space, the base being towards the gum.

In England, it has been taught that in cases where the foramen at the apex of the root is widely patent such as in immature teeth, crowns could not be satisfactorily applied, but practical experience has proved this to be an error.

You are most of you aware that I teach that a chronic abscess, in connection with a broken-down tooth, discharging itself through a fistulous opening, that shows no tendency to yield to treatment by medication will often rapidly disperse, where the canal is filled and the stump crowned and bitten on.

The probable cause of the abscess is Nature's abhorrence of an useless member, therefore the broken-down tooth acts as a source of chronic irritation to the adjacent tissues, but when crowned and bitten on it again coming into use the surrounding part is stimulated and the inflammatory products are re-absorbed.

As an instance I might mention a case communicated to me by our esteemed late House-Surgeon, Mr. Fisk.

At the beginning of last winter, Mr. Fisk, at my suggestion, crowned a broken-down tooth having a chronic abscess in connection with it, that had not shown the least improvement, after several weeks of dressing. About two weeks after crowning, the abscess had disappeared. In the summer the patient returned with a very swollen face, reporting the crown to have come away a few days previous ; it was replaced and swelling rapidly subsided. When a bicuspid or molar is broken down and has a living unexposed pulp, it will usually be best to apply an all gold cap, as then very little tooth tissue need be removed, but in such cases the pulp is very liable to die.

If the pulp be dead and there is sufficient tooth substance standing to hold the rubber-dam, apply it ; should it be very much broken down, dress off the enamel and shape the roots as for a collar, then make a band of thin metal to fit, and use this to hold the rubber-dam. The rubber-dam having been applied, open the mouth of the pulp chamber and cut away all the dentine that forms the constriction at the neck, but do not drill out the canals, the perfect reaming out of the canals is in most cases an impossibility; owing to their irregular shape and direction. Also there is a chance of perforating the side or driving septic matter through the apex, and moreover the stump is weakened.

Any part of the constriction left, acts as a scraper removing particles of debris from the bristle as it is withdrawn.

In filling the root this septic matter will be pushed to the extreme apex.

Having cut away the constriction at the neck and thus formed the canal into a funnel, remove all debris of pulp and tooth, then syringe the canals out with an antiseptic wash, Very fine bristles, roughened with a file to hold a strain of cotton, should be worked up and down the canals to thoroughly clean them.

The canals having been well washed out, should be dried and filled with a material of low conducting power, which can be easily introduced and will form a solid filling at the apex.

Gutta-percha and oxychloride of zinc are useful in filling roots.

Dr. Whitten, of Boston states that oxychloride of zinc turns any remains of pulp to leather. He introduces it on a fine bristle, at first very thin, gradually using it thicker as the root is filled until at last he introduces the pure powder.

I have modified his method in finishing by introducing a gutta-percha cone, which acts as a piston on the soft oxychloride of zinc thus filling all small spaces.

Oxychloride of zinc is very useful in cases of irregular shaped roots where it is impossible to remove the pulp thoroughly, but for ordinary cases dissolved gutta-percha cones are best.

The cones should be made as wanted, and then they will be about the size of the canal, and the full piston action is obtained.

The canals having been filled, the upstanding remains of the tooth is cut away. The greater part of this is readily removed by using a drill Messrs. Ash & Sons have made me, it is simply a long fissure bur with a chisel point.

Two holes are made with the point from the labial to lingual surface, one at the mesial, the other at the distal part of the tooth. The tissue between the holes is now cut away with the fissue part of the drill, then one blade of a pair of excising forceps is placed in the labial, and the other in the lingual opening, the handles are pressed together, and the crown comes away.

The above is the method employed in California when felling large trees.

If a crown that solely depends for its support on the dowel is to be applied, cut the stump down to a point a little beneath the gum margin, then it will not be so likely to decay by decomposition of saliva and other substances.

When a gold cap is to be used the stump may be left, standing well above the gum margin, but the sides must be made parallel.

Should a crown with a collar be applied, cut the stump away at the labio-cervical margin to a point a little beneath the gum edge. It may be left standing above the gum at the lingual surface slightly.

In order that the collar may fit the root closely all the way down, the side of the stump should be made parallel. This is best accomplished by paring off the enamel all around the stumps with chisels.

Dr. Whitten's broken back and Dr. Bennett's Nos. 5 and 6 are useful for this purpose. The enamel having been trimmed off, finish shaping the stump with a safety point shouldered fissure bur.

If the stump is shaped cone-like the collar will stretch and thus become loose every time it is removed in fitting, and it may be driven past the portion trimmed, when the projecting edge will be a source of constant irritation to the surrounding tissue. Moreover the ledge thus formed will permit of the accumulation of decomposing secretions. A dowel on no account need be of greater length than half an inch.

When using a metal-backed tooth in crowning an incisor or canine it is advisable in preparing the root for the dowel to cut as far as possible in the lingual aspect of the canal, as by this means the head of the dowel will be imbedded in the solder and thus be more strongly fixed to the cap.

When using a Logan dowel prepare the pulp canal with a conical drill similar in shape.

If the crown is to be a simple dowel crown, previous to taking an impression, shape a piece of wood like the dowel, and place this in the pulp-canal, removing it with the impression. For a gold cap or collar crown, an impression is best taken as follows:

Take a strip of telephone metal and fit it to the stump as if about to make a collar of that material. When it is roughly fitted place it upon the stump and take an impression by the patient biting into a piece of modelling composition or wax. When quite hard remove this from the mouth and cast in plaster, with the little band in position.

The following are the best metals for crowning:

Coin gold for gold cusps and bicuspid and molar bands. Green gold for incisor and canine collars.

Soft platinum for incisor and canine collar tops. Crowning metal one side gold and the other platinum for bicuspid and molar bands and backing certain teeth.

Fit coin gold bands roughly to the model obtained with the little telephone plate and then fit them in the mouth.

All fitting of green gold collars should be done in the mouth. In soldering a band or collar always have the join at the lingual part, and solder edge to edge. If the join is lapped there is always a point where it does not fit the stump.

The best clamp is a piece of binding wire. Do not mallet bands or collars on, if the stump is properly shaped and the band is the right size it will go on far better without the mallet.

In trimming a collar down at the labio-cervical margin always leave a little extra height for final driving home. The edge of a collar or band should as far as possible conform to the alveolar margin.

To make the surface of a collar flat, to which the top is to be fastened, use a flat file.

To prevent bands and collars bending in filing, fill them with composition.

In soldering on tops use as little solder as possible, place it on the outer edge and on a soldering gridiron, solder over a Bunsen flame.

The circumference of a tube tooth should be slightly larger than the cap it is to be fixed to, after fixing grind it to the same size.

The edge of a flat tooth should project just over the labio-cervical edge of the cap, but this overhanging part must be all cut away after soldering.

Back the porcelain with gold when the teeth are of a yellow shade, and with crowning metal with the platinum towards the porcelain when of a bluish tint.

Often for strength when backing a frontal or lateral tooth, it is as well to grind the porcelain away at the cutting edge to a gradual slant and carry the back up to the extreme edge. Occasionally on account of darkening the color this should not be done, but the backing cut away nearly to the pins.

In such cases a flat should not usually have been used but a tube tooth. When making a bicuspid or molar faced crown, the backing should be carried over the cutting and the cervical edges.

The pin surface of a saddle-back tooth should be ground flat with a safety edge stone, before being backed. In backing, the pins should be bent inwards as they are not in the way at the labio-cervical edge.

Specimens I exhibit will illustrate the preceding points better than a fuller description. The band to which the saddle-back tooth is soldered should be cut down to a gradual slope from the lingual surface to the labio-cervical margin with a flat file.

To obtain the cusps for a gold crown either place the band on the stump and fill it with composition or wax, which is then bitten into. The bands and contents are now removed and cusps are carved in this composition or wax, an impression of which is taken in moldine and cast in fusible metal and the die thus obtained is used to strike up the cusps; or my own method ,which I find much more expeditious is to strike up cusps in Messrs. S. S. White's dies and knock down the places bitten on with blunt punches. This avoids the unsightly appearance produced when in grinding gold cusps to the bite, the solder is exposed and thus rendered liable to discoloration.

Fill all crowns by horizontally grooving the pulp chamber and notching the dowel if one is used; dry out the canal and fill it and the interior of the crown with oxy-phosphate of a creamy consistency. Drive the crown home with a notched tooth-brush handle, and one or two light blows with a lead mallet.

When the setting is quite hard, trim away all surplus of cement with a broken-back chisel.—*Ohio Journal.*

